# Zosteriform cutaneous metastases of HER-2 positive breast carcinoma resolved after treatment with lapatinib^☆^

**DOI:** 10.1016/j.abd.2024.07.012

**Published:** 2025-01-30

**Authors:** Marcela Hernández-Coronado, Iván Alejandro Rivera-Alonso, Adrian Cuellar-Barboza, Jorge Ocampo-Candiani

**Affiliations:** Department of Dermatology, Hospital Universitario “Dr. José Eleuterio González” Universidad Autónoma de Nuevo León, Monterrey, Nuevo León, Mexico

*Dear Editor,*

Cutaneous Metastases (CM) represent 1% to 4.3% of all metastatic occurrences and 2% of all skin cancers. Breast cancer is the most common tumor associated with CM in women and usually portends a poor prognosis. Metastases can manifest with various morphological and clinical features, occasionally resembling other dermatological conditions, with nodules being the predominant presentation.^1^, ^2^ More rarely a zosteriform pattern has been described with only a few reports in the literature.^3^ Herein, we report a case of zosteriform cutaneous metastases from breast carcinoma successfully treated with lapatinib.

A 44-year-old Latin-American woman was referred from the oncology clinic for a three-week evolution dermatosis on the left thoracic wall. Five years prior, she was diagnosed with stage IIIA right breast cancer, HER2-enriched, BRCA wild-type, with metastases to the central nervous system and lymph nodes. Her treatment included a right radical mastectomy, chemotherapy with trastuzumab, pertuzumab, and docetaxel, followed by capecitabine, whole-brain radiation, and lymph node dissection. Despite undergoing extensive chemotherapy, her carcinoma spread to the left breast nine months before her dermatology consultation, leading to the initiation of a trastuzumab monotherapy regimen. Examination revealed a painful and pruritic dermatosis characterized by erythematous plaques and nodules, arranged in a dermatomal distribution on the posterolateral region of the left thorax (Fig. 1A and B). Dermoscopy of the plaques showed a pink background with polymorphous vessels (Fig. 1C). A skin biopsy was performed, and histopathology demonstrated dermal infiltration by neoplastic cell clusters dispersed among collagen fibers and infiltrating lymphatic vessels (Fig. 2, Fig. 3). These findings were consistent with a metastatic carcinoma from the breast tumor, leading to the diagnosis of zosteriform cutaneous metastases from breast carcinoma.

**Fig. 1:**
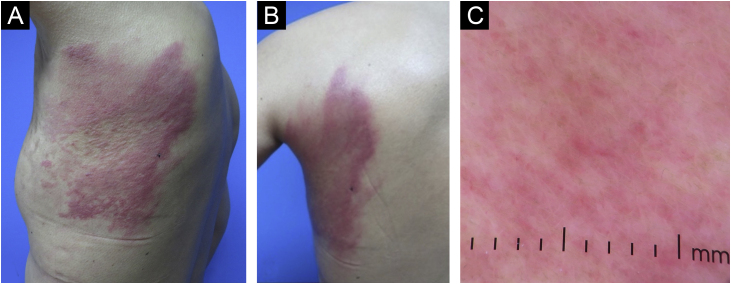
(A and B) Erythematous nodules and plaques distributed in a zosteriform pattern in the left thoracic wall and trunk, contralateral to the primary tumor. (C) Dermoscopy of zosteriform cutaneous metastases. Linear, irregular and polymorphic vessels with background erythematous structureless areas.

**Fig. 2:**
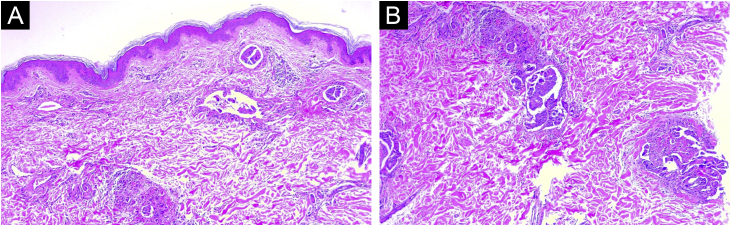
(A) Low power histological evaluation showing neoplastic cells infiltrating dermis. (Hematoxylin & eosin, 60×). (B) High-power histological evaluation showing clusters and nests of polymorphic cells with nuclear atypia and hyperchromatic nuclei forming ductal structures infiltrating blood vessels (Hematoxylin & eosin, 80×).

**Fig. 3:**
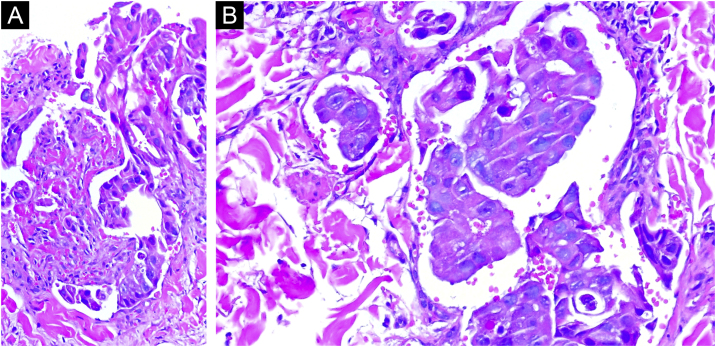
Clusters of atypical neoplastic cells arranged in nests and cords. Notably, the nuclei of these cells exhibit significant atypia in terms of size and shape, along with prominent nucleoli (Hematoxylin & eosin, 100× and 200×).

Consequently, a new monotherapy regimen with oral lapatinib 1 g/day was initiated. The skin metastases disappeared after one month of lapatinib administration, and the patient was considered to be in partial response after completing four months with this treatment (Fig. 4A and B). Dermoscopy revealed only a few structureless whitish areas with a pinkish hue (Fig. 4C). After 14 months of follow-up, no skin lesions or significant adverse effects were observed.

**Fig. 4:**
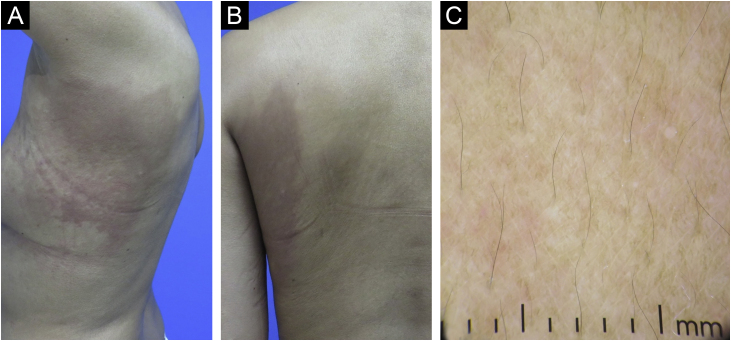
(A and B) The patient reported complete resolution of symptoms and showed remarkable improvement of the lesions one month after initiating a new therapy regimen with lapatinib 1 g/day. (C) Dermoscopy of the lesion after successful treatment. Structureless whitish areas with a pinkish hue.

A zosteriform pattern in breast carcinoma skin metastasis has been seldom described in the literature.^3^ It typically manifests as hardened lesions and may appear as papules, nodules, or pseudo-vesicles arranged in a dermatomal distribution, usually misdiagnosed as herpes zoster. The chest wall and abdomen are the predominant locations for these metastases. In more than 50% of cases, skin metastases develop ipsilateral of the primary tumor. That is not the case for our patient, where her metastases appeared on the contralateral breast. Several theories have been postulated to elucidate the pathogenesis of this uncommon distribution. These include a Koebner-like reaction occurring at the site of a previous herpes zoster infection, perineural lymphatic dissemination, and spread through the vessels associated with the dorsal root ganglion. However, the exact mechanism is still speculative.^4^ The presence of CM, particularly originating from breast adenocarcinoma, indicates a poor prognosis.^2^

There is limited evidence on the dermoscopy of cutaneous metastases from breast cancer.^5^, ^6^, ^7^ In our case, we report findings consistent with the literature with the presence of branching linear or polymorphic vessels and structureless erythematous areas. Additionally, we observed that following the clinical resolution of the lesions, dermoscopy showed a significant reduction in erythema and polymorphic vessels. Further information is needed to determine whether specific dermoscopic findings correlate with different clinical and histopathological types of cutaneous metastases from breast cancer.

Lapatinib is an oral dual kinase inhibitor targeting both Epidermal Growth Factor Receptor (EGFR) and Human Epidermal Growth Factor Receptor 2 (HER2) that has demonstrated clinical efficacy in patients with advanced or metastatic HER2+ breast cancer who have received prior therapy, including an anthracycline, a taxane, and trastuzumab.^8^ Our case demonstrates its efficacy in this clinical setting with an appropriate safety profile. A limitation in our report is the absence of a new histopathological examination of the initially affected site.

This report highlights the utilization of tyrosine kinase inhibitors in treating cutaneous metastases among patients with metastatic disease who have undergone multiple treatments. Further studies are needed to assess the safety and cost-effectiveness of these agents as monotherapy for cutaneous metastases of breast cancer.

## Authors’ contributions

Marcela Hernández-Coronado: Intellectual participation in the propaedeutic and/or therapeutic conduct of the studied cases; writing of the manuscript or critical review of important intellectual content; critical review of the literature.

Iván Alejandro Rivera-Alonso: Writing of the manuscript or critical review of important intellectual content; critical review of the literature.

Adrian Cuellar-Barboza: Intellectual participation in the propaedeutic and/or therapeutic conduct of the studied cases; writing of the manuscript or critical review of important intellectual content; final approval of the final version of the manuscript.

Jorge Ocampo-Candiani: Writing of the manuscript or critical review of important intellectual content: final approval of the final version of the manuscript.

## Financial support

None declared.

## Conflicts of interest

None declared.
